# Application of slightly acidic electrolyzed water (SAEW) for surface disinfection in waste sorting and sewage treatment plants: a pilot study

**DOI:** 10.13075/ijomeh.1896.02734

**Published:** 2026

**Authors:** Marcin Cyprowski, Bartłomiej Wróbel, Anna Ławniczek-Wałczyk

**Affiliations:** Central Institute for Labour Protections – National Research Institute, Department of Chemical, Aerosol and Biological Hazards, Laboratory of Biohazards, Warsaw, Poland

**Keywords:** slightly acidic electrolyzed water, bacteria, fungi, sewage treatment plant, waste sorting plant, surface disinfection

## Abstract

**Objectives:**

The objective of this study was to evaluate the effectiveness of surface disinfection using slightly acidic electrolyzed water (SAEW), concerning microorganisms detected in organic dust at a municipal waste sorting plant (WSP) and a sewage treatment plant (STP).

**Material and Methods:**

Five sampling sites were selected at each facility. Four disinfectants used in the tests included: SAEW solutions with active chlorine concentrations of 100 mg/l and 300 mg/l; a mixture of SAEW (100 mg/l) and silver nanoparticles (25 mg/l); and a commercially available disinfectant, a mixture of alcohols. Surface swab samples were collected to evaluate microbial contamination and compare bacterial and fungal levels after disinfection.

**Results:**

Analysis of the control samples (with no disinfectant application) revealed significant microbiological contamination of the surface tested. The tested disinfectants showed varying effectiveness against the microorganisms under study. For the aerobic and anaerobic bacteria, the highest decrease in concentrations was achieved when SAEW (300 mg/l) was applied. For the sampling sites in WSP, the log reduction values (LRV) were 2.32 and 1.99, respectively. For samples from the STP, the LRVs were 2.57 and 2.42. The most significant decrease in fungal concentration was induced by a commercial disinfectant made from a mixture of alcohols. For the samples collected at WSP, the LRV was 2.06, while for those from STP, it was 0.95. The lowest biocidal effectiveness was found for the preparation consisting of a mixture of SAEW and silver nanoparticles, for which the LRV values did not usually exceed 1.00.

**Conclusions:**

The results indicate that SAEW can be a promising alternative to the traditional alcohol-based disinfectants in occupational environments contaminated with organic dust. However, more comprehensive project should be conducted that would include a wider variety of work surfaces to be tested and a broader range of active chlorine concentrations.

## Highlights

- Biocidal effectiveness of slightly acidic electrolyzed water (SAEW) depended on the active chlorine concentration used.
- Slightly acidic electrolyzed water showed the strongest biocidal activity against bacterial microorganisms.
- Silver nanoparticles additive did not improve the disinfection efficiency of SAEW.

## INTRODUCTION

Municipal waste management and sewage treatment plants (STPs) are the major sources of organic dust which is a heterogeneous mixture of non-biological particles, such as silica, and biological particles of the plant, animal, and microbial origin. Organic dust can occur either as aerosol or settled dust gathering on the surface of construction elements of buildings and indoor equipment [1]. Settled organic dust serves as a reservoir for numerous bacterial and fungal species, some of which can be pathogenic to humans [2–4]. Exposure to organic dust accounts for various health issues in workers, including respiratory, gastrointestinal and skin diseases [5].

In workplaces where organic dust exposure can be a health hazard to workers, it is essential to implement procedures that ensure cleanliness at workstations. A study by Alonso et al. [6] showed that the disinfection of workspaces in a waste sorting plant (WSP) in Spain helped to effectively eliminate the *Coxiella burnetii* bacterium that had caused a local outbreak of the Q fever among the workers. Unfortunately, the disinfectants used in this study turned out to be highly irritating to the skin and mucosa, which points to the need for applying effective protection measures for the staff handling the disinfection tasks.

Various disinfection techniques are available, including the use of peroxide-based agents, sodium hypochlorite, ethyl alcohol, isopropanol, or γ radiation [7]. However, studies have shown that also electrolyzed oxidation water (EW), obtained by electrolysis of water solution of sodium chloride (NaCl), can serve as a disinfectant. During electrolysis, hypochlorous acid (HOCl) is generated, which exhibits a strong biocidal effect against bacteria and viruses [8]. A significant advantage of EW, compared to the previously used biocidal agents, is its low toxicity [9]. Thanks to this property, EW has become commonly used for disinfecting workspaces of different kind, e.g., in food industry [10], dental surgeries [11], and nursing homes [12]. It has also been effectively applied for disinfection at WSPs [13]. However, no information is presently available regarding EW use for disinfection of contaminated workspaces in STPs. Consequently, little is known about the biocidal effectiveness of EW used in municipal waste management, and sewage treatment facilities and further research is necessary to clarify this.

In view of the above, a pilot study was undertaken to evaluate the effectiveness of surface disinfection using slightly acidic electrolyzed water (SAEW). The assessment concerned aerobic bacteria (AEB), anaerobic bacteria (ANB), and fungi (FUN), which were detected in organic dust collected from the surface of equipment in a small WSP and a sewage treatment facility.

## MATERIAL AND METHODS

The study was conducted at a municipal WSP and a STP in a small town in central Poland, with a population of approx. 10 000. Organic dust measurements were performed in August 2025. Five sampling sites were selected at each facility. The samples were collected from the surface of equipment for wastewater treatment and the areas used for waste sorting, as these locations can accumulate organic dust. Furthermore, floor and window sill surfaces were included in the analysis due to the frequent natural contact of plant workers with these building components. Microclimate parameters were not measured at the sampling points because all were indoors, where outdoor conditions strongly influenced temperature and humidity readings. On the day of the measurements, the air temperature at 11:00 a.m. was 25°C, and the relative humidity was 55%. A comprehensive list of locations where the study disinfectants were tested is displayed in Table 1.

**Table 1.** Characteristics of sampling sites in the waste sorting plant and sewage treatment plant under study, Poland, August 2025

Facility	Sampling site
Waste sorting plant	– sorting cabin for coarse fractions >34 cm, window sill – pre-sorting cabin, floor – bag ripper, housing – bunker container, housing – recyclable materials press, housing
Sewage treatment plant	– sewage sludge press, housing – excess sludge thickener, housing – pump room, floor – heating system in the process building – screens, housing

At each sampling site, 5 square spots, 10 × 10 cm each, were designated. Control samples were collected from the surface of 1 spot, while the other 4 were treated with the prepared disinfectant solutions. The disinfectants used in the tests included:

– a SAEW solution with an active chlorine concentration (ACC) of 100 mg/l, produced in a GH-40 generator (Envirolyte Industries International Ltd., Tallin, Estonia), with mean (M) pH of 5.05 and standard deviation (SD) of 0.03;
– a SAEW solution with ACC of 300 mg/l, and pH M±SD 5.14±0.05;
– a mixture (v/v) of SAEW and ACC of 100 mg/l, combined with silver nanoparticles, of 20–30 nm in size, at a concentration of 25 mg/l (Nanografi Nanotechnology, Ankara, Turkey), with pH M±SD 6.25±0.06;
– a commercially available disinfectant, as a mixture of ethyl alcohol, isopropyl alcohol, and didecyldimethylammonium chloride (SchulkePolska Ltd., Warsaw, Poland), with pH M±SD 7.29±0.06.

Each of the prepared disinfectants was sprayed onto the surface of the 4 designated test spots using a handheld pressure sprayer, delivering approx. 5 ml of the solution. The disinfectants were left to sit for 5 min. After that, surface swab samples were collected both from the surface of the control area and 4 test areas using the “dry-wet swab” technique, in accordance with the ISO 18593:2018 standard [14]. For sampling, wet Cliniswab swabs containing 10 ml of neutralizing solution, and dry cotton swabs (Aptaca S.p.A., Canelli, Italy) were used.

The collected samples were transferred to the laboratory where they were centrifuged at 2000 revolutions per minute (rpm) on a Multi Reax shaker (Heidolph Scientific Products GmbH, Schwabach, Germany) for 5 min. Afterwards, 10-fold dilutions were prepared and plated on the following microbiological media: Tryptic Soy Agar with sheep blood for AEB, Schaedler Agar for ANB, and Malt Extract Agar, containing chloramphenicol and streptomycin, for FUN (Graso, Jabłowo, Poland). Bacterial samples were incubated at 37°C for 48 h, while fungal samples were incubated at 30°C for 5 days. After incubation, colonies were counted using Apple iPad Air (5.gen) with the BactiCALC application (Central Institute for Labour Protection – National Research Institute, Warsaw, Poland) [15]. The concentrations were calculated and expressed as log10 CFU/cm^2^.

The results were presented as M±SD for each disinfectant used over a given test area, in WSP and STP. The authors conducted t-test and ANOVA to compare the differences in bacterial and fungal concentrations after disinfection, and applied the Scheffé test, adopting p < 0.05 as significance level.

## RESULTS AND DISCUSSION

Analysis of the control samples (with no disinfectant application) revealed significant microbiological contamination of the surface tested. As for the measurements performed in WSP, the mean AEB concentration was 7.84 × 10^4^ CFU/cm² (SD = 5.25 × 10^4^ CFU/cm²), while for ANB it was 4.82 × 10^4^ CFU/cm² (SD = 3.23 × 10^4^ CFU/cm²), and for FUN 1.96 × 10² CFU/cm² (SD = 0.74 × 10² CFU/cm²). In the measurements conducted in STP, the following mean concentrations of the microbial agents were found: 3.16 × 10^4^ CFU/cm² (SD = 4.08 × 10^4^ CFU/cm²) for AEB, 1.13 × 10^4^ CFU/cm² (SD = 1.29 × 10^4^ CFU/cm²) for ANB, and 1.18 × 10² CFU/cm² (SD = 2.55 × 10² CFU/cm²) for FUN. Notably, the mean ANB and FUN concentrations showed significant differences between the facilities (p < 0.05).

Remarkably, the findings regarding ANB and FUN concentrations in WSP are in line with the results obtained by Guo et al. [13]. However, AEB concentrations at STP were found to be several times higher than those reported by Stobnicka-Kupiec et al. [16]. It is important to note that, to the best of the authors' knowledge, the contamination of work surface with ANB in STP has not yet been investigated, while respective contamination in WSP was a subject of just a few studies, including those by Cyprowski et al. [2].

**Figure 1.**
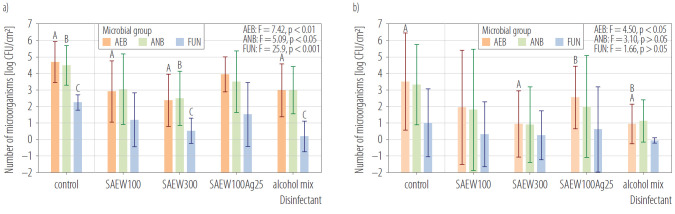
Aerobic bacteria (AEB), anaerobic bacteria (ANB) and fungi (FUN) concentrations after application of the tested disinfectants on work surface area in a) waste sorting plant (WSP) and b) sewage treatment plant (STP), Poland, August 2025

The tested disinfectants showed varying effectiveness against the microorganisms under study (Figure 1). However, their biocidal effect remained consistent across samples from both the plants. For the AEB and ANB samples, the highest decrease in concentration was achieved when SAEW solution with ACC of 300 mg/l was applied. For the sampling sites in WSP, the log reduction values (LRV) were 2.32 and 1.99, respectively. In contrast, for samples from the STP, the LRVs were 2.57 and 2.42.

A, B, C – samples for which significant differences were found. SAEW – slightly acidic electrolyzed water.

The most significant decrease in fungal concentration was induced by a commercial disinfectant made from a mixture of alcohols. For the samples collected at WSP, the LRV was 2.06, while for those from STP, it was 0.95. It would be interesting to compare the authors' findings with those of other studies on the disinfection of work surfaces using SAEW in different occupational settings. The underlying reason is the scarcity of data on this issue. The only study on a similar topic is the research conducted by Guo et al. [13] which demonstrated a maximum LRV of 1.70 for bacterial concentrations on the waste container surfaces. One should note that these authors analysed SAEW with ACC not exceeding 44.5 mg/l. Their study revealed that SAEW significantly affected the microbial diversity in municipal waste samples. This finding is crucial since municipal waste is a source of organic dust that can settle on equipment surfaces. In the pilot study, the authors did not conduct qualitative analyses of dust samples. Therefore, the authors' presume these should be incorporated in further studies and consider a larger scope of work surfaces in order to gain a more detailed understanding of the biocidal effect of SAEW.

The findings of the presently reported pilot study indicate that the biocidal effectiveness of SAEW (ACC = 300 mg/l) is comparable to that of a mixture of alcohols used as a disinfecting agent. This effectiveness may vary depending on the specific proportion of ethyl and isopropyl alcohol used in the application [17]. Unfortunately, no data are available on the use of these 2 disinfectants in waste sorting and sewage treatment environments. However, in SAEW application for dental instrument disinfection, Kameda et al. [18] demonstrated that SAEW with ACC of 200 mg/l can be as effective as ethanol at an approximate concentration of 80%.

In the authors' study, the lowest biocidal effectiveness was found for the preparation consisting of a mixture of SAEW (ACC = 100 mg/l) and silver nanoparticles (25 mg/l), for which the LRV values rarely exceeded 1.00 (except for ANB in STP, with LRV = 1.32). To this date, no similar tests using SAEW enriched with silver nanoparticles in occupational environments have been reported. Literature data mention only 1 *in vitro* study which demonstrated that a mixture of EW (ACC = 200 mg/l) with silver nanoparticles (100 mg/l) was the most effective reagent against *Salmonella* species [19]. Nevertheless, the authors' study indicated that the lower effectiveness of SAEW with silver nanoparticles, compared to both SAEW preparations without this supplementation, may have been attributed to the chemical reaction that occurred after combining the 2 components. The findings of the study by Impellitteri et al. [20] revealed that the combination of HOCl and silver nanoparticles resulted in the formation of the sparingly soluble AgCl salt. This may have significantly contributed to reducing the biocidal properties of such preparation.

## CONCLUSIONS

Due to the preliminary nature of this study, and the fact that it was conducted on a rather limited number of samples, the authors are cautious in drawing more definite conclusions. In general, the results indicate that SAEW can be a promising alternative to the traditional alcohol-based disinfectants. However, its effectiveness in reducing microbiological contamination varies based on the concentration of active chlorine used in SAEW solutions. Before any practical recommendations can be formulated, a much more comprehensive project should be conducted that would include a wider variety of work surfaces to be tested and a broader range of ACCs. Also, in-depth qualitative analyses should be performed to identify which bacterial and fungal species are most susceptible to SAEW in these occupational environments.
